# Supplementary material for: Mutations in PCYT2 disrupt etherlipid biosynthesis and cause a complex hereditary spastic paraplegia
Source: Brain. 2019 Oct 22;142(11):3382–97. doi: 10.1093/brain/awz291 (PMC6821184; doi:10.1093/brain/awz291)
Supplement: awz291_Supplementary_Data [file awz291_supplementary_data.zip › awz291-Suppl_data/Supplementary_Data4.zip!/Supplementary information 3 - fibroblast lipidomics.zip!/Fibroblast lipidomics/Patient1 vs Patient2/Statistical plots and data/TG species/Boxplots all.pdf]

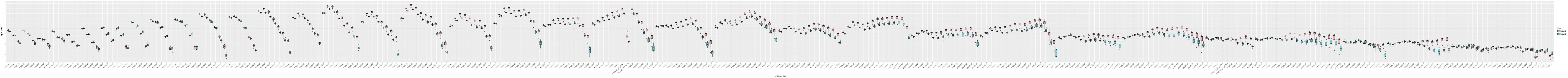
log10 value
3
2
1
0
−1
−2
TG(34:0)
TG(35:0)
TG(35:1)
TG(36:0)
TG(36:1)
TG(36:2)
TG(37:0)
TG(37:1)
TG(37:2)
TG(38:0)
TG(57:10)
TG(57:11)
TG(58:0)
TG(58:1)
TG(58:2)
TG(58:3)
TG(58:4)
TG(58:5)
TG(58:6)
TG(58:7)
TG(58:8)
TG(58:9)
TG(58:10)
TG(58:11)
TG(58:12)
TG(59:0)
TG(59:1)
TG(59:2)
TG(59:3)
TG(59:4)
TG(59:5)
TG(59:6)
TG(59:7)
TG(59:8)
TG(59:9)
TG(59:10)
TG(59:11)
TG(59:12)
TG(60:0)
TG(60:1)
TG(60:2)
TG(60:3)
TG(60:4)
TG(60:5)
TG(60:6)
TG(60:7)
TG(60:8)
lipids species
label
Patient1
Patient2
